# Temperature-Dependent and Time-Resolved Luminescence Characterization of γ-Ga_2_O_3_ Nanoparticles

**DOI:** 10.3390/nano13091445

**Published:** 2023-04-23

**Authors:** Marina García-Carrión, Julio Ramírez-Castellanos, Emilio Nogales, Bianchi Méndez

**Affiliations:** 1 Department of Materials Physics, Faculty of Physical Sciences, University Complutense of Madrid, E-28040 Madrid, Spain; 2 Department of Inorganic Chemistry, Faculty of Chemical Sciences, University Complutense of Madrid, E-28040 Madrid, Spain

**Keywords:** gallium oxide, gamma phase, nanoparticle, luminescence, decay time

## Abstract

The temperature-dependent luminescence properties of γ-Ga_2_O_3_ nanoparticles prepared by a precipitation method are investigated under steady-state and pulsed-light excitation. The main photoluminescence (PL) emission at room temperature consists of a single blue band centered around 2.76 eV, which hardly undergoes a blueshift of 0.03 eV when temperature goes down to 4 K. The emission behaves with a positive thermal quenching following an Arrhenius-type curve. The data fitting yields two non-radiative levels affecting the emission band with activation energies of 7 meV and 40 meV. On the other hand, time-resolved PL measurements have also been taken and studied as a function of the temperature. The data analysis has resulted in two lifetimes: one of 3.4 ns and the other of 32 ns at room temperature, which undergo an increase up to 4.5 ns and 65 ns at T = 4 K, respectively. Based on both stationary and dynamic PL results, a model of radiative and non-radiative levels associated with the main emission bands of γ-Ga_2_O_3_ is suggested. Finally, by using PL excitation measurements, an estimation of the bandgap and its variation with temperature between 4 K and room temperature were obtained and assessed against O’Donnell–Chen’s law. With this variation it has been possible to calculate the average of the phonon energy, resulting in 〈*ħ*ω〉 = 10 ± 1 meV.

## 1. Introduction

Gallium oxide is a wide-bandgap material with great potential in optoelectronic applications, mainly due to its ultra-wide bandgap (4.7–4.9 eV), chemical stability and polymorphous nature. In particular, five Ga_2_O_3_ crystalline phases have been reported, with β-Ga_2_O_3_ (monoclinic) being the most-stable and -studied phase. However, α (orthorhombic), γ (cubic), δ (cubic bcc), and e (hexagonal) phases, although metastable, are also under investigation since they could provide further tunability of their physical properties or an improvement in the performance of devices that use β-Ga_2_O_3_ [1,2]. Among them, the g-phase is one of the less studied, with little knowledge about its structural and physical properties so far. The crystalline structure has been reported as a distorted cubic spinel structure with a lattice parameter of 8.237 Å [3]. The energy bandgap has been theoretically estimated to be 4.4 eV for the indirect transition and 5.0 eV for the direct transition [4], with no data from experimental measurements yet reported. Some of the applications of the polymorphs of Ga_2_O_3_ are linked to photocatalysis or to solar energy conversion. For example, the photocatalytic activity of Ga_2_O_3_ nanoparticles (NPs) of several phases (α, β and γ), for the destruction of volatile aromatic pollutants in air or for water splitting, have been recently explored [5,6]. It has been reported that although β-Ga_2_O_3_ NPs would exhibit better performance due to their good crystal quality in comparison with other polymorphs, γ-Ga_2_O_3_ NPs may play an excellent role in photocatalytic applications because of their high oxygen-vacancy concentration as a consequence of the not-so-perfect crystallization of this phase [7]. Regarding solar energy conversion, the use of nanocomposites with γ-Ga_2_O_3_ NPs has been proven to be a better passivation material compared to those that embedded β-Ga_2_O_3_ NPs [8]. In this later case, the reason for this performance enhancement is still not well understood. In the aforementioned applications, there is a better knowledge of the light absorption and emission properties of γ-Ga_2_O_3_ and a better understanding of the potential improvement of their usage in devices. To that end, the investigation of optical properties related to the presence of native defects, such as the oxygen vacancies, in γ-Ga_2_O_3_ NPs is required. The literature about the optical properties of this phase is scarce. A few works have reported the photoluminescence (PL) of colloidal Ga_2_O_3_ nanocrystals of different sizes [9,10]. However, studies of time-resolved and temperature-dependent PL measurements have not been reported yet.

One of the challenges in the research of polymorphic materials is the selection of a suitable synthesis route. In particular, the synthesis of Ga_2_O_3_-based nanomaterials has been tackled under many approaches, including physical and chemical methods, to produce the desired nanomaterial [11]. In the case of Ga_2_O_3_ nanoparticles, the hydrothermal method [12], the solution combustion route [13] or the controlled precipitation route [5] are rather simple and cost-effective techniques. Among them, the precipitation route allows for a fine tuning of the microstructure by accurately controlling the pH of the solution. The method offers a valid and reliable way to synthetize several polymorphs via the simple control of the synthesis conditions, such as temperature, reaction time, pH or atmosphere. Herein, we have used the precipitation method to achieve chemically stable γ-Ga_2_O_3_ NPs. In some applications, such as in photocatalysis, as-grown material can be used directly; however, applications in optoelectronic or power electronic devices would require further processing steps to achieve suitable assemblies, as heterostructures, which become active elements in the devices.

In this work, the temperature-dependent luminescence properties of γ-Ga_2_O_3_ nanoparticles prepared by a precipitation method are investigated under steady-state and pulsed-light excitation. The analysis of the PL results obtained under stationary and dynamic conditions has allowed us to suggest some radiative and non-radiative levels associated with the main emission bands of γ-Ga_2_O_3_. In addition, by using photoluminescence excitation (PLE) measurements, an estimation of the bandgap and its variation with temperature between 4 K and room temperature was obtained for the first time and assessed against the empirical Varshni and O’Donell formulae.

## 2. Materials and Methods

In this experiment, γ-Ga_2_O_3_ NPs were prepared by a precipitate method by dissolving gallium nitrate hydrate (Ga(NO_3_)_3_ × H_2_O) in distilled water, as explained elsewhere [1]. Ammonium hydroxide solution was slowly added into that solution to obtain a basic pH (pH~8). The resultant precipitate was filtered and dried in air. The obtained powder was calcined in air at 250 °C for 14 h.

The structural characterization was carried out by means of X-ray diffraction, performed with a PANalytical X’Pert MPD (CuK_α_ irradiation, k = 1.5404 Å) system. In addition, Raman spectroscopy was conducted with a Horiba Jobin Yvon LabRAM HR800 confocal microscope. Luminescence properties were assessed by photoluminescence (PL), and PLE and time-resolved PL (TR-PL) techniques were performed in an Edinburgh Instruments FLS1000 spectrometer equipped with a continuous 450 W Xe lamp and a pulsed LED with λ_exc_ = 256.8 nm. The system was also equipped with an Oxford Instruments cryostat that allows measurements in the 4–300 K temperature range. Transmission electron microscopy (TEM) and high-resolution TEM (HRTEM) measurements were performed in a JEOL 300 at the ICTS-CNM facility.

## 3. Results

**Figure 1:**
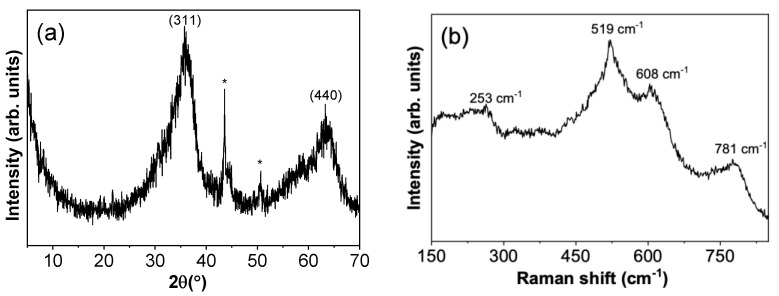
(**a**) XRD diffractogram from nanocrystalline γ-Ga_2_O_3_. Peaks labeled with an asterisks correspond to the sample holder. (**b**) Raman spectrum from γ-Ga_2_O_3_ nanocrystals using a He-Cd laser with 325 nm.

Figure 1a shows the XRD pattern of the γ-Ga_2_O_3_ nanoparticles (NPs). The maxima can be assigned to the cubic structure, space group Fd3/m, with the lattice parameter a = 8.24 Å (ICDD: 01-082-3194) of the γ-Ga_2_O_3_, in agreement with other works [4]. No peaks associated to other Ga_2_O_3_ phases are found. The broad peaks are a hint of the small size of the particles and of a not-perfect crystallization. The two peaks marked with an asterisk in the plot are associated with the material of the sample holder used for these measurements. The XRD diffractogram agrees with those reported by other authors [5] in γ-Ga_2_O_3_ NPs obtained by similar routes. The average crystal size was calculated from the values of the form factor K, the wavelength of the incident radiation λ, the full width at half maximum B of the diffraction maximum (311), and the diffraction angle θ, all of them provided by the ICDD, along with the Scherrer formula: D = K·λ/(Bcosθ). The mean value is 9.43 ± 0.02 nm. This value is slightly higher than the crystal sizes reported in the literature of γ-Ga_2_O_3_ NPs synthetized by precipitation or combustion-based methods, which are about 3–5 nm [5,13].

To further investigate the crystalline ordering, Raman spectroscopy measurements were carried out and the results are shown in Figure 1b. The Raman spectrum presents some broad peaks, as expected from the XRD diffraction characterization. In spite of that, some peaks can be resolved for the vibration modes of this phase centered at 253, 519, 608, 781 cm^−1^, possibly attributed to the tension and bending movement of the Ga-O bonds. To our knowledge, there are few Raman studies on γ-Ga_2_O_3_ NPs, and they also report broad peaks that would be related to the small average size of the nanoparticles and a weak Raman response [5,13]. Hence, it is not possible to correlate the Raman modes with specific symmetries.

**Figure 2:**
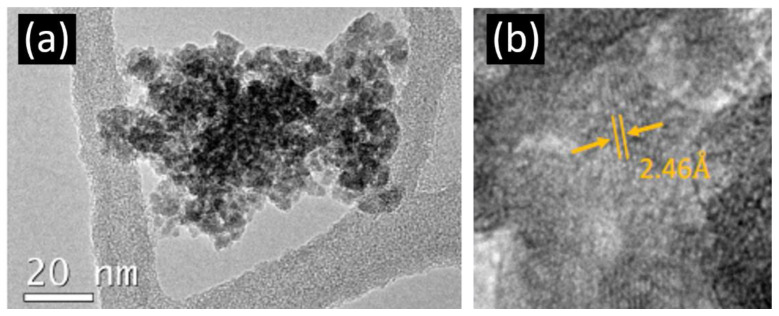
(**a**) Low-magnification TEM image of the γ-Ga_2_O_3_ nanocrystals, (**b**) HRTEM image of one nanoparticle from the agglomerate shown in (**a**).

Low-magnification TEM and HRTEM images of γ-Ga_2_O_3_ NPs are shown in Figure 2. A nanoparticle conglomerate is shown in Figure 2a, where crystallites of sizes clearly below 20 nm are observed, in agreement with the value calculated by Scherrer’s formula from X-ray diffraction. Figure 2b shows a high-resolution TEM image in which an interplanar distance of 2.46 Å was measured. This value corresponds to the XRD peak assigned to the (311) plane in Figure 1. Finally, it is worth mentioning that the TEM images suggest that part of the material is amorphous, which is consistent with the broad peaks observed both in XRD (Figure 1a) and Raman spectroscopy (Figure 1b) results.

**Figure 3:**
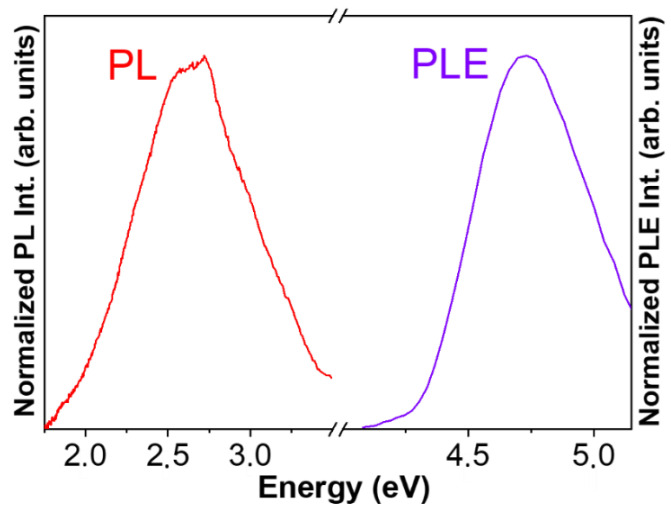
PL (red line) and PLE (purple line) spectra of γ-Ga_2_O_3_ nanoparticles acquired at RT. PL was excited with photons of 4.7 eV. PLE was acquired monitoring the emitted photons of 2.7 eV energy.

We now proceed with the study of the luminescence properties of the γ-Ga_2_O_3_ NPs. Figure 3 shows the PL and PLE spectra obtained at room temperature (RT). The PL emission spectrum was acquired with an excitation energy of 4.7 eV while the PLE excitation spectrum was obtained collecting emitted photons with a 2.7 eV emission. It is observed that the PL emission is dominated by a broad visible band, with the maximum around 2.66 eV (466 nm). This blue emission can be attributed to donor-acceptor transitions (DAP) involving deep donors and acceptors, due to the presence of intrinsic point defects such as oxygen vacancies (Vo) acting as donors and Ga vacancies or Ga–O vacancies pairs (V_Ga_, V_Ga_ – V_O_) acting as acceptor centers, which agrees with published results [14,15,16]. The rather high intensity of this visible emission would imply a high density of point defects in the nanoparticles, which is consistent with the above structural results. On the other hand, the PLE spectrum yields a band with a maximum at 4.72 eV that could be linked to the energy bandgap of the γ-Ga_2_O_3_. This broad visible emission could be of interest in the design of white light-emitter devices as an alternative to the selective rare-earth doping of Ga_2_O_3_ to simultaneously achieve red, green and blue emission lines [17].

To better understand the recombination mechanisms and the kinetics of the luminescence related to point defects in these nanoparticles, both steady-state and pulsed excitation probes have been used along with the study of both PL and PLE spectra in a range of temperatures from T = 4 K to RT. In doing so, information about the activation energies of defect-related energy levels involved in the emission and radiative and non-radiative lifetimes can be determined. In addition, the monitoring of the PLE band with temperature allows us to investigate the temperature dependence of the bandgap of γ-Ga_2_O_3_ [18,19,20].

**Figure 4:**
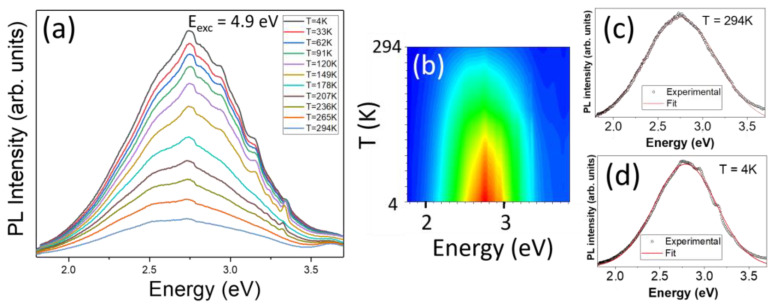
(**a**) PL spectra as a function of temperature with an excitation energy 4.9 eV. (**b**) Contour map of the integrated PL versus temperature. Deconvolution of the PL spectra at (**c**) RT and (**d**) T = 4 K.

The PL spectra series, excited with a photon energy of 4.9 eV, is displayed in Figure 4a. Figure 4b shows the corresponding contour map of the integrated PL emission as a function of the temperature. It is observed that the PL intensity increases as the temperature decreases, which means a luminescence thermal quenching. Single spectra at RT and at T = 4 K are shown in Figure 4c,d, respectively, along with their deconvolutions into Gaussian functions. In both cases, a nice deconvolution was obtained with a single band, centered at 2.79 eV at 4 K and at 2.76 eV at RT, which we will call the γ-vis band. It is worth noticing that there is almost no shift of this broad emission band as the temperature varies. On the other hand, the features appearing on the high-energy side of the emission band are regarded as artifacts, after trials under different conditions.

The PL thermal quenching of luminescence bands related to defect points is often assessed against the following empirical formula that takes into account an eventual number of non-radiative energy levels, *i*, that compete with the main radiative level [21,22]:

(1) IPL (T)=I01+∑iAi exp(−EikB T)

where *I*_0_ would be the PL intensity at very low temperature, *A_i_* are constants, and *E_i_* are the activation energies of non-radiative levels. The physical meaning of the *A_i_* constants is not quite clear and depends on the model used to understand the PL thermal quenching in each case [22]. However, in oxides with emission bands related to native defects, the coefficients *A_i_* could be related to the ratio between the luminescence radiative lifetime *τ_R_* and the non-radiative lifetime *τ_NR_* [21].

**Figure 5:**
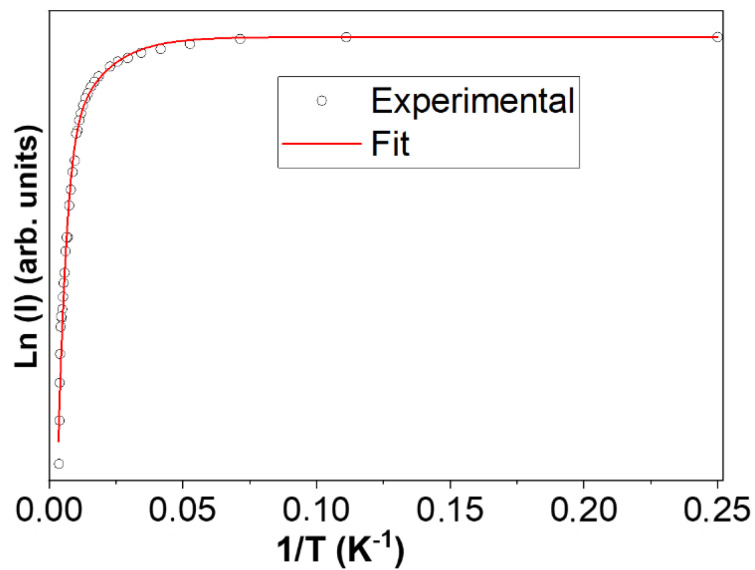
PL intensity of the γ-vis band represented as a function of 1/T. Dots are experimental data from recorded PL spectra at each temperature and red line is the result of the data fitting to Equation (1).

Figure 5 shows the evolution of the PL intensity with respect to the temperature obtained from each deconvolution of the PL spectra shown in Figure 4a along with the fitting of experimental data to Equation (1). In this case, it has been necessary to consider two non-radiative levels with activation energies *E*_1_ and *E*_2_, since otherwise the thermal excitation process saturates at low temperatures. Assuming a main radiative level, *E_R_*, as that responsible for the observed PL band, *E*_i_ stands for the difference between each non-radiative level and the radiative level, being *E*_1_ = *E_NR_*_1_ − *E_R_* and *E*_2_ = *E_NR_*_2_ − *E_R_*. The fitting results of the PL experimental data yields the values of *A*_1_ = 0.7 ± 0.2 and *E*_1_ = 7 ± 1 meV and *A*_2_ = 14 ± 3 and *E*_2_ = 40 ± 4 meV, which are summarized in Table 1. The line profile of PL variation allows us to distinguish two regions: one at low temperatures (<60 K) with an almost constant PL intensity and another one at higher temperatures (>60 K) with a sharp decrease in PL intensity. This “threshold” temperature agrees with the presence of the closest energy level (*E*_1_) that saturates at around 60 K. Even though the higher energy level E_NR2_ would require a very high temperature to be activated, both non-radiative levels could be related to surface states when taking into account the surface-to-bulk ratio in the case of nanoparticles. These states would act as traps for carriers favoring the luminescence quenching in the γ-vis emission.

**Figure 6:**
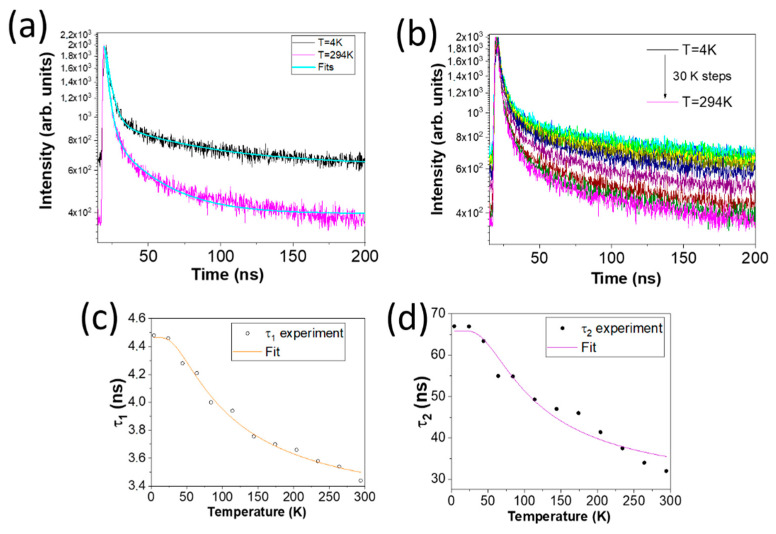
(**a**) PL intensity as a function of time at 4 K (black line), at RT (pink line) and fitting curves (blue lines) to Equation (2). (**b**) PL intensity series as a function of time used to determine the lifetime decays at 30 K temperature intervals. (**c**) *τ*_1_ (T) and (**d**) *τ*_2_ (T) calculated lifetimes (white and black dots) along with the fitting curves to Equation (3).

In order to obtain more information about the luminescence kinetics in the γ-Ga_2_O_3_ NPs, time-resolved (TR)-PL measurements were taken by exciting with a pulsed LED (λ_exc_ = 256 nm) over a range of temperatures between 4 K and RT, which allowed us to study the PL lifetime temperature dependence. Figure 6a shows the PL intensity decay at room temperature (pink line) and 4 K (black line), which show an exponential time decay. Two exponential functions, with characteristic lifetimes t_1_ and *τ*_2_, are needed to obtain an accurate matching with the experimental data, according to the following equation [23,24]:

(2) IPL t=A exp −τ1t+B exp −τ2t

Generally, the effective lifetime can be expressed as 1/*τ* = 1/*τ_R_* + 1/*τ_NR_* when taking into account both radiative and non-radiative recombination. However, when considering the thermal population of the energy levels of the emission-related center, the lifetime dependence with the temperature can be expressed as follows:

(3) 1τ=1τR+e−ΔEkBTτNR

where Δ*E* is the separation energy between the radiative and non-radiative levels, *τ*_R_ is the radiative recombination time and *τ_NR_* are the lifetime of non-radiative decays. By fitting the PL decay curves of Figure 6a to Equation (2), the two lifetimes obtained for the g-vis emission band at each temperature are: *τ*_1_ (RT) = (3.4 ± 0.1) ns and *τ*_2_ (RT) = (32 ± 4) ns, while t_1_ (4 K) = (4.5 ± 0.1) ns and *τ*_2_ (4 K) = (67 ± 2) ns. This variation of lifetime with temperature could be related to the different electron occupation of the energy levels in thermally activated processes, since the lifetime is inversely proportional to the free electrons. In our case, it can be noticed that there is a slight decrease in one process (Δ*τ*_1_ = 1.1 ns) and an increase in the second characteristic calculated lifetime (Δ*τ*_2_ = 35 ns) in the explored temperature range.

In order to better understand the temperature dependence, a series of TR-PL spectra have been recorded over a range of temperatures between 4K and RT, as represented in Figure 6b. From each curve, the values of *τ*_1_(T) and *τ*_2_(T) can be obtained and fitted to Equation (3). Figure 6c,d show the calculated lifetimes (white and black dots) of t_1_ and t_2_, respectively, along with the resulting curves from the fitting of these data to Equation (3). Table 2 summarizes the results obtained for Δ*E*, *τ_R_* and *τ_NR_* for the two lifetimes.

Based on what has been explained above from our results, the recombination center deduced from *τ*_1_(T), Δ*E*_1_, could be assigned to that one, *E*_1_, deduced from the I_PL_(T) curves. On the other hand, the second level observed by TR-PL (Δ*E*_2_) could also influence the evolution of *I_PL_*(T).

**Figure 7:**
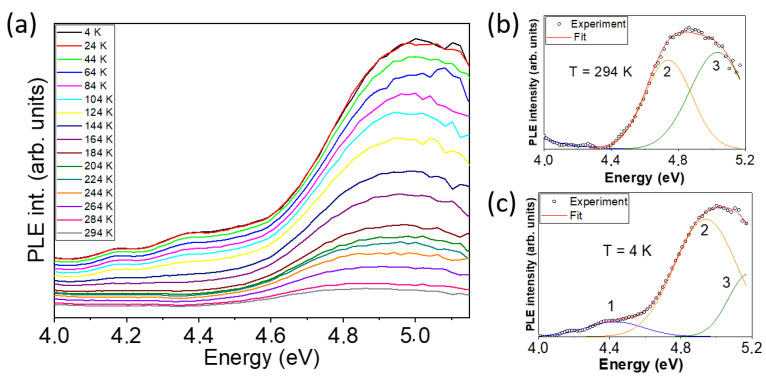
(**a**) PLE spectra as a function of temperature for an emission energy of 2.75 eV. Deconvolution of the PLE spectra (**b**) at room temperature and (**c**) at 4 K.

Finally, we study the evolution of the bandgap with the temperature for the γ-Ga_2_O_3_ NPs by means of the analysis of the PLE spectra measured at different temperatures, as presented in Figure 7a. At RT, the main observed band is centered around 4.7 eV and shifts to almost 5 eV when the temperature is lowered to 4 K. The PLE series spectra have been deconvoluted, as Figure 7b,c show for the RT and 4K cases, respectively. In doing so, three bands can be resolved. Band 1 (blue line) is possibly related to shallow donors and band 2 may represent the bandgap of the γ-Ga_2_O_3_, with a redshift from 4.93 eV at 4 K to 4.74 eV at RT. These values agreed with the only reported calculated value for the energy bandgap in γ-Ga_2_O_3_.

Several empirical formulae have been proposed to study the bandgap temperature dependence, which usually decreases as temperature increases. This is associated to the dilatation of the crystalline lattice that alters the relative positions of the valence and conduction bands. Since the thermal expansion at low temperatures is not linear, Varshni proposed a formula (Equation (4)) in which the parameter *α* represents the slope limit at very high temperatures, and the parameter *β* is related to the Debye temperature. However, the Varshni law does not fit the bandgap temperature dependence in some materials, such as diamond or SiC, which have small thermal-expansion coefficients and the electron-phonon interaction would be the main factor affecting the bandgap shift with the temperature. O’Donnell and Chen proposed an alternative expression (Equation (5)) that usually renders a better fit to experimental results, in which *S* is a nondimensional constant related to the strength of the electron–phonon interaction and 〈*ħ*ω〉 is an average of phonon energy [25]:

(4) $$E_{g}\left(T\right)=E_{g}\left(0\right)-\frac{\alpha T^{2}}{\beta +T}$$

(5) $$E_{g}\left(T\right)=E_{g}\left(0\right)-S\;\langle \hbar \omega \rangle \left[coth\left(\frac{\langle \hbar \omega \rangle }{2k_{B}T}\right)-1\right]$$

**Figure 8:**
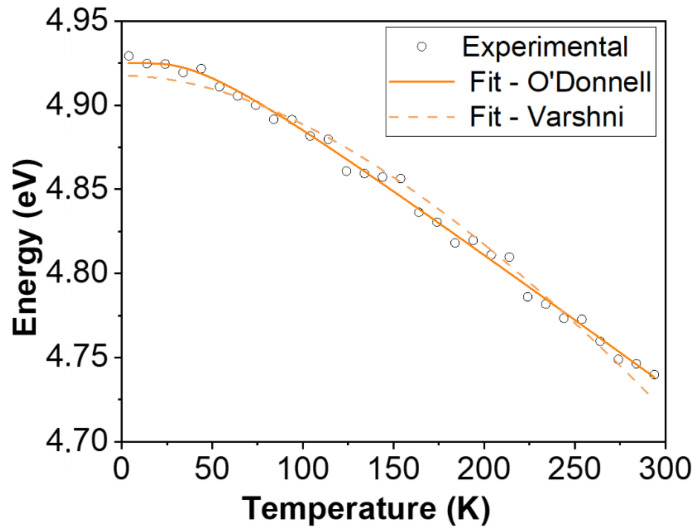
Variation in the estimated bandgap with temperature from PLE results. Open dots are calculated data—the continuous line is the fitting to the O’Donnell formula, whereas the dashed line is the fit to the Varshni equation.

Figure 8 shows the bandgap values calculated from the fitting of the PLE spectra at different temperatures (open dots) as well as the fitting curve of these data to the O’Donnell and Chen equation (continuous line) and to the Varshni equation (dashed line). The parameters resulting in the Varshni model are *α* = 0.19 meV/K and *β* = 558 ± 20 K. The *β* parameter has been reported for the case of the nanoparticles of γ-Ga_2_O_3_, giving an approximate value of 580 K [10], which is similar to that obtained here. On the other hand, the values of 〈*ħ*ω〉 = 10 ± 1 meV, *S* = (4.59 ± 0.12) × 10^−4^ and *E_g_*(0) = 4.74 eV are obtained from the equation proposed by O’Donnell and Chen. No previous works have been found on the *S* parameter nor on the average phonon energy in γ-Ga_2_O_3_. Herein, it can be observed that the O’Donnell and Chen model matches rather better than the Varshni model with our calculated results. This is consistent with the low thermal-expansion coefficients of Ga_2_O_3_ that were reported for the *β*-phase so far [26].

## 4. Conclusions

In the present work, the luminescence properties of γ-Ga_2_O_3_ nanocrystals obtained by a precipitation method are presented and discussed. It should be mentioned that hardly any studies have been found on the luminescent properties of this metastable phase of gallium oxide. Through XRD and Raman measurements, it has been verified that the nanoparticles obtained at a temperature of 250 °C exhibit the γ-Ga_2_O_3_ phase, as well as the fact that there is no presence of another phase, although some amorphous material remains in the product. In spite of that, the Raman spectra show some vibration modes of the crystallized phase. The PL study has included measurements under steady conditions as well as under a pulsed excitation source. Furthermore, both of them have been carried out over a temperature range from 4 K to RT. The main PL emission is a single band centered around 2.76 eV at room temperature, which hardly shifts when temperature decreases. The origin of this emission could be DAP transitions that involved native defects of γ-Ga_2_O_3_, as is usual in wide-bandgap oxides. The PL intensity as a function of the temperature has been fitted to an Arrhenius-type law, leading to the appearance of two temperature behavior regimes, as a consequence of the non-radiative levels affecting the emission band. These two levels have an activation energy of 7 meV and 40 meV. On the other hand, TR-PL measurements as a function of the temperature have allowed us to determine the lifetimes that control the quantum efficiency and their temperature dependence. The fitting to exponential decays of the PL intensity with time has resulted in two lifetimes: one of 3.4 ns and the other of 32 ns at RT. From the analysis of the shortest one with the temperature, a model of radiative and non-radiative levels associated with the main emission bands of γ-Ga_2_O_3_ is proposed, which agrees with the results raised from the Arrhenius fitting of the PL intensity with the temperature. Finally, the bandgap and its variation with temperature between 4 K and room temperature were obtained using PLE spectra, giving rise to a difference of 0.2 eV. The results show a rather good match to the O’Donnell formula, and a value for the average of the phonon energy, resulting in 〈*ħ*ω〉 = 10 ± 1 meV, has been deduced.

## Figures and Tables

**Table 1:** Fitting parameters of the PL thermal quenching to Equation (1).

Energy Level	Activation Energy E_i_ (meV)	Constant
E_1_	7 ± 1	0.7 ± 0.2
E_2_	40 ± 4	14 ± 3

**Table 2:** Data obtained from the temperature-dependence lifetime study.

*τ*_1_			*τ*_2_		
***τ*_R1_ (ns)**	***τ_NR_*_1_ (ns)**	**Δ*E*_1_ (meV)**	***τ_R_*_2_ (ns)**	***τ_NR_*_2_ (ns)**	**Δ*E*_2_ (meV)**
11 ± 1	4.5 ± 0.1	10 ± 1	66 ± 2	44 ± 9	15 ± 2

## Data Availability

The data that support the findings of this study are available from the corresponding author upon reasonable request.
